# Low Clinical Burden of 2009 Pandemic Influenza A (H1N1) Infection during Pregnancy on the Island of La Réunion

**DOI:** 10.1371/journal.pone.0010896

**Published:** 2010-05-28

**Authors:** Patrick Gérardin, Rachid El Amrani, Béatrice Cyrille, Marc Gabrièle, Philippe Guillermin, Malik Boukerrou, Brahim Boumahni, Hanitra Randrianaivo, Arnaud Winer, Jean-Fabien Rouanet, Michel Bohrer, Marie-Christine Jaffar-Bandjee, Pierre-Yves Robillard, Georges Barau, Alain Michault

**Affiliations:** 1 Neonatal and Pediatric Intensive Care Unit, Centre Hospitalier Régional (CHR), Saint Pierre, La Réunion, France; 2 Centre for Clinical Investigation–Clinical Epidemiology (CIC–EC) of La Réunion (INSERM/CHR/URMLR), Saint Pierre, La Réunion, France; 3 UMR S953, “Epidemiological Research on Perinatal Health and Women's and Children's Health” (INSERM/Assistance Publique des Hôpitaux de Paris), Paris, France; 4 Obstetric and Gynecology Unit, CHR, Saint Pierre, La Réunion, France; 5 Fetopathology Unit, CHR, Saint Pierre, La Réunion, France; 6 Intensive Care Unit, CHR, Saint Pierre, La Réunion, France; 7 Department of Medical Information, CHR, Saint Pierre, La Réunion, France; 8 Department of Medical Information, CHR, Saint Denis, La Réunion, France; 9 Microbiology, CHR, Saint Denis, La Réunion, France; 10 Microbiology, CHR, Saint Pierre, La Réunion, France; Columbia University, United States of America

## Abstract

**Background:**

Pregnant women have been identified as a group at risk, both for respiratory complications than for the admissions to the Intensive Care Unit (ICU) during the 2009 H1N1 influenza pandemic (pdm). The purpose of this prospective register-based cohort-study was to characterize the clinical virulence of the pdm (H1N1/09)v during pregnancy in La Réunion.

**Methods/Principal Findings:**

Over a twelve-week pdm wave (13 July to 3 October 2009), 294 pregnant women presented with an influenza-like illness (ILI) to one of the three maternity departments of the South Reunion area, Indian Ocean. Out of these, 278 were checked by RT-PCR for influenza viruses (157 positive and 121 negative, of whom, 141 with pdm flu and 132 with ILIs of non pdm origin, 5 untyped). The median body temperature was higher in women experiencing pdm flu than in those with non pdm ILI (38.9°C *versus* 38.3°C, *P*<0.0001), without evidence linked to circulating viremia. Oseltamivir was given for 86% of pdm flu cases in a median time inferior than 48 hrs (range 0–7 days). The hospitalization rate for pdm flu was of 60% and not associated with underlying conditions. Six viral pneumonia and fourteen asthma attacks were observed among 84 hospitalized pdm flu cases, of whom, only one led to the ICU for an acute lung injury. No maternal death occurred during the pdm wave. None adverse pregnancy outcome was associated with pdm flu. No congenital birth defect, nor early-onset neonatal influenza infection was attributable to pdm flu exposure.

**Conclusions/Significance:**

This report mitigates substantially the presumed severity of pandemic H1N1/09 influenza infection during pregnancy. The reasons for which the clinical burden of H1N1/09 influenza virus may differ worldwide raise questions about a differential local viral-strain effect and public health preparedness, notably in timely access to special care and antiviral treatments.

## Introduction

Since the beginning of the first third-millennium pandemic, foremost was the concern that 2009 triple-reassortant pandemic (pdm) H1N1 influenza (H1N1/09) virus could be more life-threatening than its seasonal peers [1], as evidenced by its unusual clinical burden in young adults [2]–[6], who experienced more than half of Intensive Care Unit (ICU) stays and deaths [6]–[9], mainly due to the acute respiratory distress syndrome (ARDS) complicating pneumonia of viral origin [4], [7]–[9].

In this context suggesting enhanced virulence for this novel influenza strain, further supported by alveolar damage in animal models reminiscent of highly pathogenic H5N1 avian viruses [10]–[12], pregnant women, who exhibited higher case-fatality-ratios (CFR) in past influenza pandemics [13], [14], but also increased admission rates to hospital in seasonal flu [15], have been stressed as a high-risk population for pdm influenza [16]. Indeed, out of thirteen states of the USA, between 15 April and 16 June, admission rates to hospital for pregnant women were four-fold those of the US community. By the same period, among the 45 deaths notified to the US Centers of Disease Control and Prevention (CDC), six were observed during gestation, highlighting pregnant woman eligibility to a fatal issue [17]. Additionally, in the USA, pregnant women for whom oseltamivir was delayed beyond 48 hrs carried a four-fold risk to be admitted in the ICU and a 30-fold risk to die [18], whilst the high cause-specific maternal CFR anticipated that 2009 H1N1 influenza could increase the 2009 maternal mortality ratio in the country [19]. Noteworthy, similar figures were found in the south hemisphere, where pdm flu was shown increasing asthma, diabetes mellitus and preterm labor during pregnancy in Australia, whereas very preterm births, stillbirths and infant deaths heralded also adverse perinatal outcomes [20], [21]. However, Lim et al., in an Asian perspective contrasted the pregnant woman vulnerability to pdm flu in suggesting that early presentation, diagnosis and treatment could mitigate its presumed severity [22].

In La Réunion island, a French overseas department located in Mascarene Archipelago (810,000 inhabitants, southwestern side of the Indian Ocean), the first confirmed case of H1N1/09 influenza was diagnosed on 5 July (week 27), in a traveler back from Australia [23]. The human-to-human indigenous transmission was declared by our regional surveillance system, the CIRE Reunion-Mayotte (Cellule Inter-régionale d'Epidémiologie) of the French Institute of Public Health and Surveillance (Institut national de Veille Sanitaire, InVS), on week 30 (20 to 26 July) [23]. This alert was made as part of enhanced influenza surveillance, implemented soon after the first reported cases in the USA and anticipating the flu season which started with the beginning of the austral winter in July [24].

In order to characterize the spectrum of pdm (H1N1/09)v throughout pregnancy and get a clear picture of its clinical burden in pregnant women to provide unbiased information for risk management, we designed an inception hospitalized-based cohort study from a regional birth register, representative of the reproductive population [25]. The main objective of our cohort was to compare the clinical virulence of pdm flu during pregnancy with that of influenza-like illnesses (ILIs) of non pdm origin (pdm ILIs), using the most complete set of indicators of clinical virulence. Additional aims included benchmarking the attack rate in pregnant women to that of the non pregnant population, the proportion of severe respiratory complications in pregnant women and non pregnant women of childbearing age.

## Methods

### Study location and participants

Our prospective study took place in the three south Reunion maternity departments (5,300 deliveries per year for a population of 300,000 inhabitants): the two public level-3 and level-1 maternity wards of the Groupe Hospitalier Sud-Réunion (GHSR), the largest hospital on the island (standard care comparable to that available in Europe, 4,100 deliveries per year), plus those of a collaborative private level-1 maternity (1,200 deliveries per year) [25].

We enrolled all pregnant women, parturient women and their offspring referred for consultation or admitted at the maternity departments between 5 July 2009 (date of the first pdm influenza infection confirmed on the island) and 3 October 2009 (fall of the outbreak). To minimize the rate of paucisymptomatic cases and the possibility of underascertainment, general practitioners (GPs) and ambulatory midwives were also invited to check flu symptoms, using the REPERE (Réseau Périnatal Réunion) network mailing list, as well as to refer their patients to the hospital for virological testing.

### Ethical considerations

For the use of the data, oral consent was obtained from each patient or a first-degree relative, as the investigations were carried out under the standard care procedure for this infection, in accordance with the dedicated amendment recommendations of the Committee for Clinical Research of the GHSR which approved specifically this observational study. In France, a written consent is mandatory only if the medical acts or the products used are not standard for the diagnosis, the treatment or the monitoring (art. 88-II, law 2004-806, JO, 08/11/2004; art. 31-I, law 2006-450, JO, 04/19/2006) [25]. The information was given by a senior physician in face to face, in French or in Creole with the help of a translator when necessary. This included a brief presentation of the study, the terms of virological testing and antiviral treatment, as indicated by the French College of Gynecologists and Obstetricians (CNGOF) [26], as well as the return of RT-PCR result in a sealed envelope.

### Data Collection and Screening

Midwives and nurses collected full obstetric history from maternity booklets and additional questioning in the framework of our daily epidemiologic perinatal survey [25]. These includes comorbidities, the commonest flu symptoms: rhinorrhea, conjunctivitis, sore throat, cough, shortness of breath, headache, backache, muscle ache, joint pain, fever, nausea, vomiting, diarrhea and asthenia, enabling to redefine ILI in accordance with its widely used definition [17].

For symptomatic women, whether presenting for examination at the outpatient clinic or for hospitalization in the obstetrical unit, one-step TaqMan RT-PCR assays were performed from nasopharyngeal swabs, serum, placenta, or amniotic fluid samples using the Light Cycler 2.0 system or Light Cycler 480 (Roche Diagnostics). For the women referred late in the course of their flu, diagnosis of influenza was conducted retrospectively using serum complement fixation testing. This technique enables the detection of antibodies specific to influenza A or B antigens. A four-fold rise of antibodies titers against A or B antigens in paired sera or a titer at least of 1∶80 (five dilutions) were considered to define recent influenza with accuracy [27].

### Case ascertainment

An ILI was defined in presence of at least one of the common flu symptoms listed above. A confirmed case of pdm influenza was defined as an ILI with positive A(H1N1/09)v RT-PCR. A probable case of pdm flu was defined as an ILI with positive influenza A, but negative H1 and H3 for seasonal variants test, or with a positive A serology. A confirmed or probable case of pdm influenza was referred as pdm flu. A case of ILI without criteria of pdm flu, or diagnosed as seasonal flu, was referred as non pdm ILI.

An additional group referred as controls accounted for non ILI pregnant women who gave birth in June 2009, the preceding month the influenza occurrence. Finally, a subsidiary group was extracted from hospital statistics and constituted by ILIs in non pregnant women of reproductive age (15–44 yrs) assessed in the Emergency Department (ED) during the influenza season without formal evaluation of (H1N1/09)v status.

Pneumonia was defined for an acute respiratory illness with audible crackles and chest infiltrates on X-ray films. Acute Lung Injury (ALI)/ARDS were defined in presence of bilateral hypoxemic pneumonia requiring FiO_2_/PaO_2_ indexes between 200 and 300 or less than 200 Torr, respectively.

In the absence of earlier infection, maternal ILI was classified as ante partum in women whose clinical signs had occurred between the conception and the seventh day preceding labor (d-7) to account for a seven-day maximal length of influenza viral shedding under oseltamivir [28]. Maternal ILI was classified as per partum when symptoms occurred between d-7 and d+7 around delivery with a concomitant positive RT-PCR, and post partum for any women presenting symptoms beyond d+7.

Exposed neonates were checked twelve hours after birth by RT-PCR assay using nasopharyngeal and/or oral swabs. For the neonates born from a symptomatic mother in per partum or in early post partum, additional RT-PCR tests were performed on serum at day 0 and/or during the first week of life.

### Management of pregnant women

None of the non pharmaceutical measures addressed to mitigate the incidence and severity rates of pdm influenza in the community was fully applied in La Réunion, as part of a public health priority to protect the pregnant woman, beyond the fact that healthcare providers (HCPs) were invited to treat pregnant women separately, preferentially as outpatients, using surgical masks and alcoholic solutions, as advocated by the CNGOF [26].

At household level, oseltamivir (75 mg once a day for 10 d) was given to the relatives to avoid household transmission, as the epidemic occurred before the availability of vaccines in France.

At hospital level, high and low viral density areas were drawn, and HCPs wore FFP2 or surgical masks and used alcoholic solutions to avoid transmission to the pregnant women in maternity setting. In case of fever, women were checked to rule out alternative diagnoses such as pyelonephritis, chorioamniotitis, listeriosis, dengue and chikungunya. After RT-PCR testing, women were treated using oseltamivir (75 mg two times *per* d for five d) and quarantined until negativation of nasal swab, in accordance to CNGOF recommendations [26].

### Statistical Methods

To estimate the regional attack rate in pregnant women, we derived the cumulative incidence rate (CIR) observed in the framework of the South Reunion perinatal survey to the whole island territory, assuming *T_b_*, the overall regional births as a constant (*T_b_*≈11,000), the number of births in the south per nine month as a constant (*S_b_*≈4,100) and a proportional factor, the *k* factor (*k* = *T_b_*∶*S_b_* = 2,68). We then compared the CIR in pregnant women to the figure provided by the surveillance system for the community [29], after removing the pregnancies-specific burden.

Clinical cases were classified as mild or severe influenza (pneumonia, ALI/ARDS, ICU admission). Preterm labor (PTL) was defined in presence of painful regular uterine contractions and cervical changes occurred between 22 and 37 wks of amenorrhea.

Adverse pregnancy outcomes included preterm premature rupture of membranes (PPROM), gestational diabetes mellitus (GDM) and pregnancy-induced hypertension (PIH), most gestation issues (miscarriage, stillbirth, preterm live birth, PTLB) and the mode of delivery. Neonatal outcomes gathered congenital malformations (EUROCAT, European surveillance for Congenital Abnormalities definition [30]), intra-uterine growth (Oken charts [31]), fetal heart abnormalities during labor, amniotic fluid quality, one-minute Apgar score, neonatal survival and early-onset neonatal influenza infections.

Pandemic flu cases were compared with non pdm ILIs in terms of circulating viremia, clinical presentation, severe influenza respiratory complications, adverse pregnancy and neonatal outcomes, as indicators of clinical virulence.

Hospitalized pdm flu pregnant women were roughly compared to the non pregnant women of reproductive age in terms of pneumonia and in terms of admission to the ICU.

Pregnant women with pdm flu or with non pdm ILI were subsequently compared with all the control pregnant peers, assuming as neglected the possibility that asymptomatic forms would change significantly the relationship with pregnancy issues in flu cases.

Exposed neonates born from infected mothers were compared with unexposed controls on congenital malformations, growth, amniotic fluid, fetal heart rate, one-minute Apgar score and survival.

Proportions were compared using Chi square or Fisher exact tests as appropriate. Continuous variables were compared using Student or Mann-Whitney tests as appropriate. Risk ratios (RRs) were calculated with standard methods and 95% confidence intervals (95% CI) using Taylor series. All analyses were computed in Stata (release 9; StataCorp. 2005, Texas, USA). Statistical significance was set at *P* = 0.05 for all analyses, except for those of adverse pregnancy and neonatal outcomes for which a Bonferroni correction was applied at *P* = 0.01 to account for multiple comparisons.

## Results

**Figure 1:**
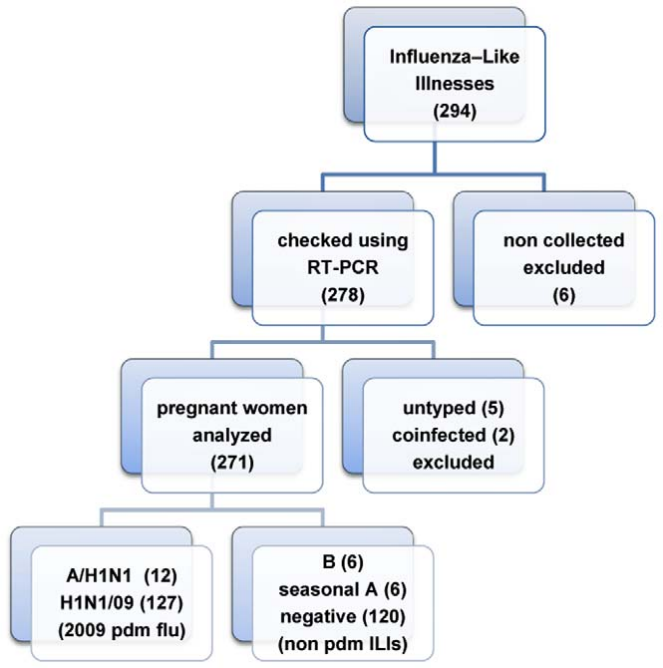
Flow chart of pregnant women with flu symptoms, maternity department, Saint-Pierre, Reunion Island, 2009.

During the twelve-wk of the pdm wave in La Réunion, which lasted for pregnant women from July 13 (wk 29) to October 3 (wk 40), out of 4,100 estimated ongoing pregnancies, 294 (7%) pregnant women were referred to a flu-dedicated consultation in one of the three South Reunion maternity departments, representing nearly half of the obstetrical visits during that period (45%, 294/653 passages). Out of these, 278 (95%) were checked for influenza viruses using a Pasteur specific RT-PCR assay (Table S1). The distribution of the pregnant women ascertained for flu symptoms in South Reunion maternities throughout the 2009 pdm wave is presented in Fig. 1.

**Figure 2:**
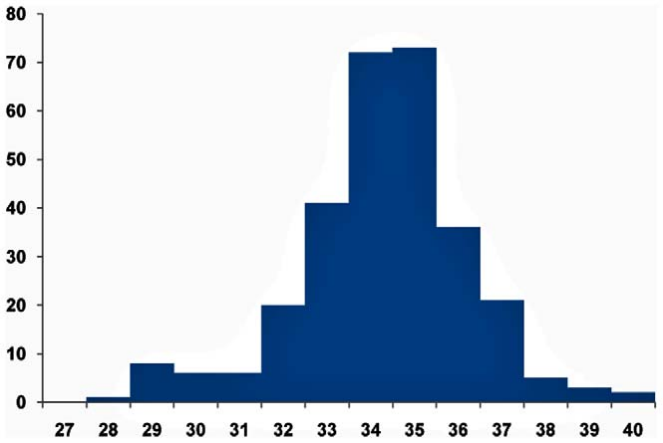
Pregnant women with flu symptoms, maternity department, Saint-Pierre, Reunion Island, 2009. Weeks, from 5 July (w28) to 3 October (w40).

**Figure 3:**
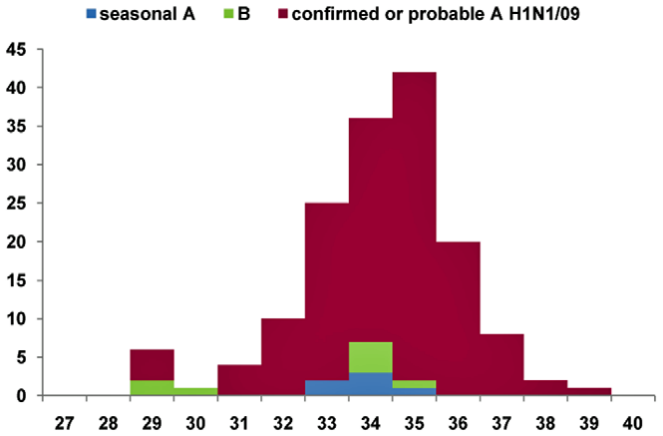
Pregnant women with confirmed influenza virus, maternity department, Saint-Pierre, Reunion Island, 2009. Weeks, from 5 July (w28) to 3 October (w40).

Weekly case numbers of maternal ILIs and pdm H1N1/09 influenza are displayed on separate epidemic curves in Fig. 2 and Fig. 3. The CIR of probable or confirmed cases of pdm H1N1/09 flu derived from south Reunion birth register was estimated at 3.6% (95%CI: 3.3%–3.9%), leading to a regional cumulative case estimate during pregnancy of 392 cases, or 3,568 cases per 100,000 pregnant women (95%CI: 3,267–3,868). In comparison, the attack rate for the non pregnant community, once entrenched the pregnancies-specific burden (392 cases/11,000 estimated ongoing pregnancies), was 12.8% (relative risk reduction for pregnant women: 0.75, 95%CI: 0.72–0.77).

The timing of ILI onset through pregnancy is presented in Table S1. Out of the 251 women of ante partum onset (126 with pdm flu and 125 with non pdm ILI), none of the six confirmed pdm flu assessed by serum RT-PCR had detectable viremia, irrespective of the time of ILI from mid- to late pregnancy, precluding the possibility that severe third-trimester flu could be attributable to viremia. In the same manner, out of the 27 ILIs of per partum onset (13 with pdm flu, 14 with non pdm ILI), none of the fifteen parturients for whom such specimen was collected (10 with pdm flu, 5 with non pdm ILI), exhibited the molecular signature of influenza genome in serum, excluding vertical mother-to-child transmission at birth via a placental breakdown or a microtransfusion from a highly viremic maternal blood. Importantly, placenta (n = 22) or amniotic fluid (n = 7) specimens were RT-PCR negative arguing the uncertainty of transmission through placental or “all mucosal” routes.

None of the pregnant women declared a previous immunization, since H1N1/09 vaccines were not yet available in the southern hemisphere and seasonal flu and pneumococcal vaccines not recommended for pregnant women at the time of our study. Baseline characteristics and previously recognized risk factors for severe pdm flu (origin, obesity, smoking, asthma) did not differ according to the origin of flu in pregnant women (Table 1).

**Table 1:** Characteristics of pregnant women (PW) with 2009 pdm H1N1-v infection or with influenza-like illness (ILI) unrelated to 2009 pdm H1N1-v, uninfected pregnant women and non pregnant women of childbearing age (15–44 years) with ILI of indistinct origin, Saint – Pierre, Reunion Island, 5 July to 3 October 2009.

	PW with 2009 pdm flu		PW with non pdm ILI		*P* value*	Uninfected PW		*P* value**	Non PW with indistinct ILI		*P* value***
	(n = 139)§		(n = 131)			(n = 440)			(n = 108)§§		
**Maternal age (years)**					0.1368			0.0023			0.0005
<18	12	(8.6)	6	(4.6)		26	(5.9)		17	(15.7)	
18 – 29	96	(69.1)	89	(67.9)		241	(54.7)		58	(53.7)	
30 – 39	30	(21.6)	30	(22.9)		149	(33.9)		21	(19.4)	
≥40	1	(0.7)	6	(4.6)		24	(5.5)		12	(11.1)	
**Place of Birth**					0.5316			0.0189			0.1842
La Réunion	113	(81.3)	107	(81.7)		363	(82.5)		62	(81.6)	
Mainland France	22	(15.8)	18	(13.7)		37	(8.4)		7	(9.2)	
Other neighboring islands	3	(2.2)	6	(4.6)		38	(8.6)		7	(9.2)	
Others	1	(0.7)	0			2	(0.5)		0		
**Parity**					0.8844			0.5186			0.0806
Nullipara	48	(34.5)	48	(36.7)		168	(38.2)			(49.4)	
Primipara	47	(33.8)	45	(34.3)		123	(27.9)			(18.5)	
Multipara	44	(31.7)	38	(29.0)		149	(33.9)			(32.1)	
**Weeks pregnant at infection**					0.7658			NA			NA
0 – 13	28	(20.1)	31	(23.7)		–					
14 – 28	56	(40.3)	49	(37.4)		–			–		
>29	55	(39.6)	51	(38.9)		–			–		
**Overweight/obesity** †					0.2491			0.51			0.0004
Yes	67	(48.2)	54	(41.2)		186	(45.4)		15	(25.3)	
No	72	(51.8)	77	(58.8)		224	(54.6)		26	(74.7)	
**Smoking**					0.4660			0.1329			0.6433
Yes	27	(19.4)	21	(16.0)		56	(12.7)			(15.0)	
No	112	(80.6)	110	(84.0)		384	(87.3)		-	87.0)	
**History of current asthma**					0.1402			NA			0.0067
Yes, currently receiving drugs	8	(5.8)	2	(1.5)		–			14	(13.1)	
Yes, not currently receiving drugs	8	(5.8)	5	(3.8)		–			5	(4.7)	
No	123	(89.4)	124	(94.7)		–			88	(82.2)	

Data are numbers (and percentages in parentheses); pdm flu *vs* non pdm ILI (line 1* line 2* column 1* column 2);

**pdm flu vs non pdm ILI vs uninfected peers ([L1+L2]*L3*C1*C2*C3); **pdm flu vs non pdm ILI vs uninfected peers ([L1+L2]*L3*C1*C2*C3);

† body mass index ≥25 kg/m2;

§ 127 PCR+ confirmed H1N1/09 infections, 12 probable H1N1/09 infections (11 PCR+, 1 CF serology+), two H1N1/09 with influenza B virus coinfections excluded;

§§ Data retrieved from medical informatic charts, completed by a telephonic survey, missing data excluded from calculation

The median duration of symptoms from onset to flu-dedicated consultation was two days, irrespective of diagnosis. The clinical features of pdm flu and non pdm ILIs are depicted in Table 2.

**Table 2:** Clinical features of 2009 pdm H1N1-v infection and of influenza-like illnesses (ILI) unrelated to 2009 pdm H1N1-v, pregnant women, Saint – Pierre, Reunion Island, 5 July to 3 October 2009.

	2009 pdm flu		non pdm ILI		*P* value
Clinical symptoms	(n = 139)§		(n = 132)		
Fever above 37.8°C	127	(91.4)	93	(70.5)	<0.0001
Rhinorrhea	100	(71.9)	105	(79.5)	0.1449
Conjunctivitis	4	(2.9)	9	(6.8)	0.1292
Sore throat	83	(59.7)	87	(65.9)	0.2916
Cough	126	(90.6)	120	(90.9)	0.9407
Shortness of breath	19	(13.7)	20	(15.2)	0.9824
Backache	43	(30.9)	36	(27.3)	0.5072
Muscle ache	47	(33.8)	35	(26.5)	0.1911
Joint pain	41	(29.5)	25	(18.9)	0.0430
Nausea	19	(13.7)	13	(9.8)	0.3299
Vomiting	14	(10.1)	12	(9.1)	0.7840
Diarrhea	2	(1.4)	4	(3.0)	0.4372
Headache	15	(10.8)	12	(9.1)	0.6404
Asthenia	26	(18.7)	19	(14.4)	0.3405
Fever plus either cough or sore throat †	115	(83.3)	83	(62.3)	0.0002

Data are numbers (and percentages in parentheses) or medians (and Q_1_–Q_3_, the inter quartile range);

† defining influenza-like-illness for Jamieson et al. [17];

§ 127 PCR+ confirmed H1N1/09 infections, 12 probable H1N1/09 infections (11 PCR+, 1 CF serology+), two H1N1/09 with influenza B virus coinfections excluded; *P* value set at 0.05 for statistical significance.

The body temperature peak was higher in pdm flu than in non pdm ILIs (38.9°C vs 38.3°C, P<0.0001), but the length of fever (48 hrs) was indistinct between the two groups. Pdm flu cases were more likely febrile than non pdm ILIs. Accordingly, they fulfilled better the definition of ILI used in the USA (fever plus either cough or sore throat [17]) than non pdm ILIs (Table 2).

The leukocyte count was lower in pdm flu than in non pdm ILI. The biologic parameters characterizing pdm flu and non pdm ILI are displayed in Table 3.

**Table 3:** Biological features of 2009 pdm H1N1-v infection and of influenza-like illnesses (ILI) unrelated to 2009 pdm H1N1-v, pregnant women, Saint – Pierre, Reunion Island, 5 July to 3 October 2009.

	2009 pdm flu		non pdm ILI		*P* value
	(n = 112)§		(n = 88)		
Leukocyte count (x 10^3^ per µL)	7.5	(5.9 – 9.0)	8.8	(6.7–10.9)	0.0012
< 4,000	4	(3.6)	0		0.0021
4,000 – 10,000	90	(80.4)	56	(65.1)	
>10,000	18	(16.0)	30	(34.9)	
Neutrophil count (x 10^3^ per µL)	5.7	(4.3–7.2)	6.2	(5.1–8.8)	0.1548
< 1,500	2	(1.8)	0		0.0359
1,500 – 8,500	95	(84.8)	60	(74.1)	
>8,500	15	(13.4)	21	(25.9)	
Lymphocyte count (x 10^3^ per µL)	0.9	(0.6–1.3)	1.0	(0.7–1.5)	0.3283
< 750	39	(34.8)	25	(30.9)	0.2535
750 – 1,500	56	(50.0)	36	(44.4)	
>1,500	17	(15.2)	20	(24.7)	
Platelet count (x 10^3^ per µL)	209	(174–264)	221	(186–272)	0.9669
ASAT (U/L)	21	(16–30)	18	(15–23)	0.2598
ALAT (U/L)	14	(10–19)	12	(9–15)	0.1734
Creatinine (µmol/L)	48	(42–56)	47	(41–56)	0.5524
Potassium (mmol/L)	3.8	(3.6–4.0)	3.9	(3.7–4.1)	0.2654
C-Reactive Protein level (mg/L)	26.0	(14.7–46.0)	22.4	(12.0–36.6)	0.3861
< 25	54	(49.1)	50	(56.8)	0.2112
25–50	35	(31.8)	29	(33.0)	
> 50	21	(19.1)	9	(33.0)	

Data are numbers (and percentages in parentheses) or median (and Q_1_–Q_3_, the inter quartile range);

§ 103 PCR+ confirmed H1N1/09 infections, 9 probable H1N1/09 infections (8 PCR+, 1 CF serology+), two H1N1/09 with influenza B virus coinfections excluded; *P* value set at 0.05 for statistical significance.

Oseltamivir was given in 218 (80%) out of the 271 women compared for flu symptoms (86% for pdm *vs* 75% for non pdm ILl, *P* = 0.02), which was indicative of inappropriate use in a third. The median delay to start oseltamivir from the onset of symptoms was less than two days in both groups. The median time for apyrexia was two days in both groups and was not shortened by oseltamivir.

The overall ratio of hospitalized to ambulatory cases was 1.1 (52% to 47%). The admission rate to the obstetrical unit was higher in pdm flu than in non pdm ILIs (60% *vs* 48%, crude RR 1.3, 95%CI: 1.0–1.6, *P = *0.04) and tended to correlate with the stage of gestation (65% in third, 62% in second, 43% in first trimester, *P = *0.09). Extrapolating this admission rate on a regional basis led to estimate the hospitalization rate for pdm flu at 2% of the pregnant women (95%CI: 1.8%–2.3%). The principal motives for admission to the obstetrical unit with pdm flu were protracted fever requiring monitoring and ruling out other diagnoses (69%), PTL (14%), decreased fetal movements or fetal heart abnormalities (10%). Taken together, in pdm flu, underlying conditions and comorbidities (obesity, smoking, asthma, GDM, PIH), were not more frequent in inpatients than in outpatients (53% *vs* 44%, *P = *0.25). The median length of stay (LOS) for pdm flu was three days (range 6 hrs–12 d).

Importantly, severe respiratory complications were observed exclusively with H1N1/09 influenza. Six cases of pneumonia were diagnosed with pdm flu (crude RR: 2.0, 95%CI: 1.7–2.3, *P = *0.03), which represented barely 7% of hospitalized women (median LOS: five d, range 2–7 d). No ARDS case was noted, however seven cases of pdm flu required oxygen therapy (Six of whom treated in obstetrical unit with high-concentration bags, one ALI case admitted to the ICU for a 4-d mechanical ventilation). Fourteen patients used β2 mimetics for asthma symptoms (Table S2). The risk factors for severe pdm influenza (defined as pneumonia or severe asthma) are presented in Table 4.

**Table 4:** Risk factors for severe pandemic influenza, pregnant women, Saint – Pierre, Reunion Island, 5 July to 3 October 2009.

	Severe 2009 pdm flu		Mild 2009 pdm flu		*P* value
	(n = 10)§		(n = 129)		
**Indigenous to Indian Ocean ^a^**	9	(90.0)		(82.9)	1
**Overweight/Obesity** † **^b^**	5	(50.0)	62	(48.1)	1
**Smoking ^c^**	1	(10.0)	26	(20.2)	0.6867
**Asthma ^d^**	2	(20.0)	14	(10.9)	0.3231
Delay to start oseltamivir (hrs)	62.7	(61.7)	41.3	(27.2)	0.0524
At least one of these conditions ^a+b+c+d^	9	(90.0)	68	(52.7)	0.0425
Two conditions	0		14	(10.9)	NA
Three conditions	0		2	(1.6)	NA

Data are numbers (and percentages in parentheses) or means (and standard error);

† body mass index ≥25 kg/m^2^;

§ defined as pneumonia or severe asthma requiring oxygen; *P* value set at 0.05 for statistical significance.

Compared to non pregnant women of reproductive age (15–44 yrs) seen in the ED, hospitalized pdm flu pregnant women carried not an excess risk of pneumonia (6/84 *vs* 7/109, crude RR: 1.1, 95% CI: 0.5–2.0), or to be admitted to the ICU (1/84 *vs* 4/109, crude RR: 0.4, 95%CI: 0.0–2.6).

Overall, pregnancies with flu were twofold more likely to experience PTL than uninfected pregnant controls (crude RR 2.2, 95%CI: 1.4–3.2, *P = *0.0001). Importantly, uterine contractility associated with flu was mild and controlled by usual means for tocolysis (bed rest, calcium channels blockers, salbutamol). Hence, only one seasonal flu case led to impending PTLB. Gestational diabetes mellitus tended to occur more frequently within pdm flu than in uninfected pregnancies (13% *vs* 7%, crude RR 1.7, 95%CI: 1.0–2.6, P = 0.03). However, half of the GDM cases were diagnosed after the onset of pdm flu. After careful exclusion of alternative causes, none miscarriage or stillbirth was attributable to pdm flu. None other adverse pregnancy outcome was significantly associated with influenza (Table 5).

**Table 5:** Obstetrical outcomes associated with 2009 pdm H1N1-v infection or with influenza-like illnesses (ILI) unrelated to 2009 pdm H1N1-v or with uninfected controls, pregnant women, Saint – Pierre, Reunion Island, 5 July to 3 October 2009.

	2009 pdm flu		non pdm ILI		*P* value*	Controls		*P* value**
	(n = 139)§		(n = 132)			(n = 445)		
**Preterm labor**					0.3202			0.0007
Yes, concomitant to flu	13	(11.3)	8	(80.9)		-		
Yes, unrelated to flu	9	(7.8)	14	(12.1)		38	(8.9)	
No	93	(80.9)	93	(80.9)		392	(91.2)	
**Preterm premature rupture of membranes**					1			0.1257
Yes, concomitant to flu	1	(0.9)	1	(0.9)		-		
Yes, unrelated to flu	1	(0.9)	2	(1.7)		3	(0.7)	
No	113	(98.2)	112	(97.4)		427	(99.3)	
**Gestational diabetes mellitus**					0.0647			0.0327
Yes, after onset of flu	8	(7.0)	4	(3.5)		-		
Yes, preexistent to flu	7	(6.1)	1	(8.7)		30	(7.0)	
No	100	(86.9)	110	(87.8)		400	(93.0)	
**Pregnancy-induced hypertension**					0.5387			0.7031
Yes, after onset of flu	7	(6.1)	4	(3.5)		-		
Yes, preexistent to flu	0		1	(8.8)		17	(4.0)	
No	108	(93.9)	110	(87.7)		383	(96.0)	
**Pregnancy issues** §					0.08			0.0668
Miscarriage†	0		2	(1.7)		10	(2.2)	
Stillbirth ‡	0		4	(3.5)		5	(1.1)	
Very preterm livebirth (<33 wks)	3	(2.6)	2	(1.7)		6	(1.4)	
Late preterm livebirth (33 to 36 wks)	14	(12.2)	10	(8.7)		28	(6.3)	
Term delivery precipitated by flu	5	(4.3)	9	(7.8)		-		
Term delivery unchanged by flu	93	(80.9)	88	(76.5)		396	(89.0)	
**Mode of delivery** ¶					0.5773			0.7485
Vaginal	93	(80.9)	97	(84.3)		362	(84.1)	
Caesarian section	21	(19.1)	18	(15.7)		68	(15.9)	
Delivery expected in the forthcoming weeks¶,§	24		17			-		

Data are numbers (and percentages in parentheses);

*pdm flu *vs* non pdm ILI (line 1* line 2* column 1* column 2);

**pdm flu vs non pdm ILI vs uninfected peers ([L1+L2]*L3*C1*C2*C3);

¶ miscarriages and ongoing pregnancies excluded (censoring at 12 April 2010);

§ ongoing pregnancies excluded;

† fetal demise <16 wks;

‡ early, late ante and intra partum fetal death ≥16 wks;

§ 127 PCR+ confirmed H1N1/09.

Four congenital birth defects were diagnosed in neonates born from mother reporting ILI during pregnancy, one in the pdm flu group (ventricular septal defect, n = 1), three in the non pdm ILI group (cleft lip without cleft palate, n = 1, cleft lip with cleft palate, n = 1, hypospadias, n = 1). After careful examination of the chronology of ILI onset with respect to embryogenesis, none malformation was attributable to fever or influenza. At time of labor, parturient women experiencing concomitant flu did not exhibit more fetal heart abnormalities, meconium-stained fluid, low one-minute Apgar score than uninfected peers. None adverse neonatal outcome was associated with influenza (Table 6).

**Table 6:** Neonatal outcomes associated with 2009 pdm H1N1-v or with influenza-like illnesses (ILI) unrelated to 2009 pdm H1N1-v exposures or with unexposed controls, Saint – Pierre, Reunion Island, 5 July to 3 October 2009.

	2009 pdm flu		Non pdm ILI		*P* value*	Controls		*P* value**
	(n = 142)§		(n = 130)			(n = 440)		
**Time of exposure** ¶					0.6125	-		NA
Ante partum (< D-7)	129	(90.8)	115	(88.5)		-		
Per partum (from D-7 to D+7)	13	(8.2)	14	(10.7)				
Post partum (> D+7)	0		0	(0.8)				
**Congenital malformations** ^†,^ ¶					0.6125			
Yes, subsequent to flu	0		0			-		
Yes, preexisting to flu	1	(0.9)	3	(2.6)		5	(1.1)	
No	116	(99.1)	112	(97.4)		435	(98.9)	
**Small for Gestational age** ‡,¶					0.8040			0.478
Yes	14	(12.0)	15	(13.0)		42	(9.5)	
No	103	(88.0)	100	(87.0)		398	(90.5)	
**One-minute Apgar score** ¶					1			0.5694
<7	4	(3.4)	3	(2.6)		22	(5.0)	
7 to 10	113	(96.6)	112	(97.4)		418	(95.0)	
**Neonatal issue** ¶					0.2155			0.5112
Death	0		1	(0.9)		5	(1.1)	
Discharged from neonatal unit	8	(6.8)	3	(2.6)		21	(4.8)	
Discharged from nursery	109	(93.2)	111	(96.5)		414	(94.1)	
Expected (pregnancy on course) ¶,§	25		15			-		

Data are numbers (and percentages in parentheses); pdm flu *vs* non pdm ILI (line 1* line 2* column 1* column 2);

**pdm flu vs non pdm ILI vs uninfected peers ([L1+L2]*L3*C1*C2*C3);

¶ expected births, miscarriage and stillbirths excluded (censoring at 12 April 2010);

§ ongoing pregnancies excluded;^ †^ EUROCAT definition [30];

‡ small for gestational age neonates are defined under 10^th^ percentile using Oken growth charts [31];

§ 127 PCR+ confirmed H1N1/09 infections, 12 probable H1N1/09 infections (11 PCR+, 1 CF serology+), two H1N1/09 with influenza B virus coinfections excluded; *P* value set at 0.01 for statistical significance (Bonferroni correction).

The event of a mother-to-child vertical transmission of pdm influenza virus was sought for eleven neonates exposed long before birth, and for eleven neonates from mothers newly infected around delivery (Table S3). No evidence of early-onset clinical infection was found in the first week of life. One neonate was found a healthy carrier of H1N1/09v soon after birth, presumably owing to maternal post partum contamination. He was a small-for gestational age neonate delivered from a previously healthy woman by cesarean section for fetal heart decelerations after 24 hours of maternal fever due to pdm influenza. Maternal and infant nasal swabs returned positive at d0. Maternal viremia and placental specimen were negative. The infant remained asymptomatic apart from hypoglycemia placed on the account of growth restriction. He was treated with oseltamivir for five days without side-effects. His RT-PCR control at d7 was negative.

During the twelve-wk pdm wave, only two neonates were referred late in neonatal care unit for mild symptoms (at d17 and d21 respectively), due to household transmission.

## Discussion

This hospitalized-based cohort, designed from a well-tested regional representative birth register [25], identified all pregnant women referred to the South Reunion Island maternities for ILI or any complaint compatible with flu throughout the 2009 pdm influenza wave, which occurred in La Réunion, between July 13 (wk 29) and October 3 (wk 40), during the austral winter season. Therefore, we were able to accurately determine the clinical burden of non pdm ILI and pdm influenza during pregnancy on a regional basis. Our findings, acquired without major selection bias nor critical strain on the healthcare supply, mitigate historical and recent disturbing data on severe pdm influenza in pregnant women highlighting increased risks for hospitalization or admission to the ICU [4], [6], [13]–[21]. Interestingly, this current overview provides some arguments to demonstrate the low clinical virulence of pdm influenza (H1N1/09)v, in a context of a public health response focused on the prompt identification of clinical cases and managing clusters of infections with widespread use of anti-influenza drugs.

First, despite extensive research, we did not find any evidence for a circulating viremia that could have explained the peculiar severity of 2009 pdm influenza in pregnant women, gradually as the gestation progresses. Hence, circulating viremia, which is scarcely observed with seasonal flu [32], would have been a hallmark of clinical virulence, reminiscent of highly pathogenic H5N1 avian flu [33]. The absence of circulating viremia in such susceptible human group is in agreement with small animal models and a non-human primate model for which no evidence of viremia have been reported [10]–[12].

Second, the clinical presentation of laboratory-confirmed 2009 pdm influenza was not substantially different from that of non pdm ILIs, beyond the fact that it was more febrile and symptomatic. The latter is congruent with the seminal description of swine flu in humans [34], and consistent with the absence of previous herd immunity against this triple-reassortant virus. Noteworthy, vomiting and diarrhea, which were highlighted indicators of severity, both in ferrets and in humans [10], [11], [34], were very infrequent in our cohort. Similarly, shortness of breath, previously described as harbinger of poor respiratory compliance in US pregnant women with 2009 pdm influenza [17], was indistinct of pdm flu or non pdm ILI. Furthermore, leukocyte count was lower in women with pdm flu than in those with non pdm ILI, and consisted almost invariably in lymphopenia. However, marked lymphopenia (<750 per cubic millimeter) was not associated with severe illness. Severe lymphopenia, which is predictive of fatal ARDS in H5N1 avian flu [35], has also been found indicative of severe 2009 pdm influenza [36]. Like in seasonal influenza, its mechanism would involve apoptosis via the fas-fasL pathway [37].

Third, severe viral pneumonia was very scarce in La Réunion and contrasted sharply with the critical illness and the excess of mortality due to ARDS encountered in pregnant women in the rest of the world, evidenced by their overrepresentation in ICUs and the deaths reported in the USA [5], [18], [19], Manitoba, Canada [6], [7], Australasia [4], [9], [21], or mainland France [38]. Nonetheless, in our setting, pdm flu was associated with more frequent pneumonia or severe asthma, as witnessed heightened needs for oxygen rescue therapy. This finding should remind the primary respiratory involvement of emerging influenza A viruses, otherwise illustrated by alveolar damage in animal models [10]–[12], ventilatory or ECMO supports in critically-ill young adults [2]–[9], whose corollary, the access to ICU beds have represented a major issue over the pandemic [4], [39]. The basis of severe respiratory involvement observed in pdm influenza during pregnancy may be related to the conjunction of immunologic and physiologic changes, including the switch from cell-mediated to antibody-mediated immune responses, respiratory compromise by mechanical effect, and heightened cardiopulmonary demand to compensate the raised blood volume [17]–[21]. The paucity of ALI/ARDS cases observed in La Réunion may probably be blamed to the awareness of local HCPs, as evidenced by the prompt access to medical care. Indeed, early and easy access to antiviral treatments may have reduced the progression towards more severe disease and hereafter the admission rate to the ICU and mortality [5]–[8]. The efficacy of oseltamivir in preventing pneumonia and other influenza-related complications has long been controversial in adults and poorly investigated in high-risk groups [40], [41]. The neuraminidase inhibitors action might be larger than expected, both exhibiting antiviral and antipneumococcal synergistic properties, limiting the sialidase activity of *Streptococcus pneumoniae* [42], which may have contributed to contain pneumococcal disease during the pandemic.

Fourth, the ability of pdm H1N1/09 influenza to disrupt the natural course of gestation is not confirmed by our study. Certainly, like other investigators [20], we observed more PTL among pregnancies accompanied by pdm flu (14% *vs* 8% in uninfected controls). However, uterine contractility was mild and well-controlled by tocolytics. Subsequently, the annual incidence of PTLB (10%) was unchanged in South Reunion. As PTB was related to untractable pneumonia in previous influenza pandemics [13], [14], [16], we may explain the absence of impact of pdm (H1N1/09)v on PTLB by the low proportion (4.2%) and benignity of pneumonia in our setting. To reach a 1.8% significant discrepancy in PTLB rates between pdm flu and uninfected controls (Table 5) with a 3.4% pdm flu CIR (α = 0.05, 1–β = 0.8), it would have required enrolling over 1,800 infected pregnant women in our cohort. Similarly, at Réunion island (where the baseline GDM rate has reached 7% for a couple of years [43]), pdm influenza (H1N1/09)v was linked to heightened GDM rate like recently observed in Victoria, Australia [20]. However, while there, a cross-sectional study design did not allow to conclude on the chronological relationship between GDM and pdm flu (and vice versa), our cohort reveals that in half the cases GDM occurred before pdm flu, in half the cases pdm flu had preceded GDM, which argues definitely against a causal relationship between the two conditions. Nevertheless, the fact that year 2009 was associated with the record of GDM registered in our database (7.5%) raises questions, even if during the same time, overweight and obesity, its common determinants, have grown steadily (+2%, from 34% to 36%, from 14% to 16%, respectively).

Five, pdm influenza (H1N1/09)v infection was neither associated with a significant rise of congenital malformations, nor with early-onset neonatal influenza infections. The effects of maternal influenza on the fetus, whether due to fever or to a transplacental transmission of the virus are poorly understood [16]. The crossing of the placental barrier by a microbial pathogen is an essential indicator of virulence [44]. To date, except with highly pathogenic H5N1 avian strains [45], the placental transmission of influenza viruses lacks of evidence [16]. In this, our study adds molecular proof to the large seroepidemiological study of Irving et al. who did not find such aptitude with seasonal flu [46]. Importantly, except one healthy carrier of probably post partum transmission of maternal origin, we observed no early-onset neonatal influenza infection. This suggests more the harmlessness of pdm influenza (H1N1/09)v in the newborn than the full effectiveness of barrier measures used against its horizontal transmission. This is consistent with the paucity of neonatal infections during the pandemic.

Our study has some strengths and limitations. On the one hand, using a cohort-study design in the framework of a birth register within a public health context of awareness allowing caregivers not to be reluctant to entrust their patients to hospital, has certainly made possible a nearly exhaustive review of ILIs during pregnancy. This assertion is made plausible by the fact that the systematic examination of parturient women in the maternity department, sometimes combined with complement fixation testing, have detected only two new clinical cases among over 2,800 women who delivered in the six months following the pdm wave. We could thereby accurately determine the burden of H1N1/09 influenza without significant underreporting bias, nor concurrent overestimation of severe influenza respiratory complications. On the other hand, the low critical mass of ongoing pregnancies on the island of La Réunion may have failed to properly investigate the ratio of severe to mild H1N1/09 influenza disease.

Indeed, with only 11,000 ongoing pregnancies, a crude estimate of influenza-related critical illness in Australasia about 1∶4,300 gestations (66 ICU stays for 285,000 ongoing pregnancies), we were unable to show the role of pregnancy as a risk factor for influenza-related critical illness in La Réunion [18], [21]. Similarly, an excess of other rare events, such as congenital malformations or fetal infection, could not be detected to a large scale in our setting, unless teratogenicity or vertical transplacental transmission with pdm influenza or oseltamivir is very frequent, which has never been evidenced so far [16], [47]. Nonetheless, with only six cases of pneumonia and no ARDS for 84 laboratory-confirmed pdm H1N1/09 influenza infections admitted to the hospital (one pneumonia per 14 hospitalized infections), we were below the expected range for hospitalized pneumonia (1∶4 to 1∶2) and outside those of ARDS/pneumonia admitted to the ICU previously found in pdm flu (1∶14 to 1∶2) [17], [19], [20]. Therefore, we were unable to calculate the ARDS/infections ratio, which has been proposed as indicator of clinical virulence [39]. Nevertheless, it must be stressed that we enrolled only clinical cases in our cohort. The knowledge of the asymptomatic/symptomatic ratio in pregnant women would have provided another clue upon the clinical virulence in this population, that being attenuated in case of predominant inapparent forms. A recent study in the French adult population of reproductive age (20–39 yrs), confronting data from serosurveys in pregnant women to those of the Sentinelles Network, estimated at 4∶1 this ratio [48].

In conclusion, this report from La Réunion Island, which was representative of the population of the pregnant women in the area, mitigates substantially the severity of pdm H1N1/09 influenza infection during pregnancy. Whether its findings proceed from local physician skill and good preparedness, or from a low clinical virulence of the virus in the region, raises concerns at the time when public health agencies and policymakers draw up the balance of the pandemic. Finally, phylogenetic studies of triple-reassortant pdm influenza virus strains linked to clinical descriptions are warranted to better understand the reasons for which the clinical burden of H1N1/09 influenza virus may differ worldwide.

## Supporting Information

Table S1 RT-PCR specimens out of 278 pregnant women with an influenza-like illness (ILI) of ante-, per- or post-partum onset, Saint-Pierre, Reunion Island, 5 July to 3 October 2009.
(0.08 MB RTF)

Table S2 Respiratory complications and special care associated with 2009 pdm H1N1-v infection or with influenza-like illnesses (ILI) unrelated to 2009 pdm H1N1-v, pregnant women, Saint - Pierre, Reunion Island, 5 July to 3 October 2009.
(0.07 MB RTF)

Table S3 RT-PCR specimens for influenza-like illnesses (ILI) and 2009 pandemic flu exposures of ante-, per- or post-partum onset, offspring, Saint-Pierre, Reunion Island, 5 July to 3 October 2009.
(0.09 MB RTF)
