# Supplementary material for: Low Clinical Burden of 2009 Pandemic Influenza A (H1N1) Infection during Pregnancy on the Island of La Réunion
Source: PLoS One. 2010 May 28;5(5):e10896. doi: 10.1371/journal.pone.0010896 (PMC2878351; doi:10.1371/journal.pone.0010896)
Supplement: Table S1 — RT-PCR specimens out of 278 pregnant women with an influenza-like illness (ILI) of ante-, per- or post-partum onset, Saint-Pierre, Reunion Island, 5 July to 3 October 2009. (0.08 MB RTF) [file pone.0010896.s001.rtf]

Table S1. RT-PCR specimens out of 278 pregnant women with an influenza-like illness (ILI) of ante-, per- or post-partum onset, Saint-Pierre, Reunion Island, 5 July to 3 October 2009	
Ante partum onset	Per partum onset	Post partum onset		
(n = 251)	(n = 27)	(n = 0)	Total	
cavum + / serum -	(6)	cavum + / serum -	(11)	 cavum + / serum -	(0)	(17)	
cavum +	  (136)	cavum + 	(4)	cavum +	(0)	(140)	
cavum - / serum -	(2)	cavum - / serum -	(4)	cavum - / serum -	(0)	(6)	
cavum - 	(107)	cavum -	(8)	cavum -	(0)	(115)	
placenta -	(10)	placenta -	(12)	placenta -	(0)	(22)	
amniotic - 	(2)	amniotic - 	(5)	amniotic - 	(0)	(7)	
Total	(263)		(44)		(0)	307	
*152 positive RT-PCR specimen: 140 H1N1/09)v (of whom 2 coinfected with B), 6 B, 6 seasonal A, 5 positive untyped; 120 negative; 1 FC  serology A positive.	
