# Supplementary material for: Low Clinical Burden of 2009 Pandemic Influenza A (H1N1) Infection during Pregnancy on the Island of La Réunion
Source: PLoS One. 2010 May 28;5(5):e10896. doi: 10.1371/journal.pone.0010896 (PMC2878351; doi:10.1371/journal.pone.0010896)
Supplement: Table S2 — Respiratory complications and special care associated with 2009 pdm H1N1-v infection or with influenza-like illnesses (ILI) unrelated to 2009 pdm H1N1-v, pregnant women, Saint - Pierre, Reunion Island, 5 July to 3 October 2009. (0.07 MB RTF) [file pone.0010896.s002.rtf]

Table S2. Respiratory complications and special care associated with 2009 pdm H1N1-v infection or with influenza-like illnesses (ILI) unrelated to 2009 pdm H1N1-v, pregnant women, Saint – Pierre, Reunion Island, 5 July to 3 October 2009	
	2009 pdm flu	non pdm ILI	P value	
	(n = 139)§	(n = 132)		
Pneumonia					0.0298	
Yes	6 	(4.5)	      0			
No	133 	(95.5)	132	(100)		
Acute respiratory failure †					0.4534	
Discontinued oxygen therapy or â2 mimetics	19	(13.7)	14	(10.6)		
ALI / ARDS *	1 	(0.7)	      0			
No	119 	(85.6)	118 	(89.4)		
Need for special care counseling ‡					NA	
Cardiologist	2 	(0.1)	1 	(0.8)		
Pulmonologist	1 	(0.1)	1 	(0.8)		
Intensivist	1 	(0.1)	0			
None	136 	(99.7)	130 	(98.4)		
Hospitalization > 2 days					0.1299	
Yes	45 	(32.4)	32	(24.2)		
No	39 	(28.0)	32	(24.2)		
Outpatients	55 	(39.6)	68 	(51.6)		
Admission to ICU					NA	
Yes	1		          0			
No	0		 0			
 Data are numbers (and percentages in parentheses); †Acute lung injury or acute respiratory distress syndromes are defined by  
 hypoxemia and bilateral chest infiltrates on x-rays, with 200 < FiO2/PaO2 < 300 Torr (ALI), FiO2/PaO2 < 200 Torr (ARDS); for the case 
 of ALI, the diagnosis was made using complement fixation serology;‡Advices may be combined; § 127 PCR+ confirmed H1N1/09 
 infections, 12 probable H1N1/09 infections (11 PCR+, 1 CF serology+), two H1N1/09 with influenza B virus coinfections excluded; 
 P  value set at 0.05 for statistical significance.	
