# Supplementary material for: Low Clinical Burden of 2009 Pandemic Influenza A (H1N1) Infection during Pregnancy on the Island of La Réunion
Source: PLoS One. 2010 May 28;5(5):e10896. doi: 10.1371/journal.pone.0010896 (PMC2878351; doi:10.1371/journal.pone.0010896)
Supplement: Table S3 — RT-PCR specimens for influenza-like illnesses (ILI) and 2009 pandemic flu exposures of ante-, per- or post-partum onset, offspring, Saint-Pierre, Reunion Island, 5 July to 3 October 2009. (0.09 MB RTF) [file pone.0010896.s003.rtf]

Table S3. RT-PCR specimens for influenza-like illnesses (ILI) and 2009 pandemic flu exposures of ante-, per- or post-partum onset, offspring, Saint-Pierre, Reunion Island, 5 July to 3 October 2009 	
Ante partum ILIs 	Per partum ILIs	Post partum ILIs		
(n = 119)	(n = 14)	(n = 0)	Total	
cavum - / serum -	(5)	cavum - / serum -	(7)	cavum - / serum -	(0)	(12)	
cavum -	(3)	cavum -	(3)	cavum -	(0)	(6)	
Total	(8)		(10)		(0)	(18)	
Ante partum 2009 pdm flu	Per partum 2009 pdm flu	Post partum 2009 pdm flu		
(n = 130)	(n = 13*)	(n = 0)	Total	
cavum + / serum -	(0)	cavum + / serum -	(1)	cavum + / serum -	(0)	(1)	
cavum - / serum -	(3)	cavum - / serum -	(8)	cavum - / serum -	(0)	(11)	
cavum -	(8)	cavum -	(2)	cavum -	(0)	(10)	
oropharynx +	(0)	oropharynx +	(1)	oropharynx +	(0)	(1)	
oropharynx -	(0)	oropharynx -	(3)	oropharynx -	(0)	(3)	
gastric fluid - 	(2)	gastric fluid - 	(4)	gastric fluid - 	(0)	(6)	
Total	(11)		(21)		(0)	(32)	
*Eleven neonates collected out of 13, four collected fourfold (cavum/serum/oropharynx/gastric fluid)	
